# Identification of Histone H3 (*HH3*) Genes in *Gossypium hirsutum* Revealed Diverse Expression During Ovule Development and Stress Responses

**DOI:** 10.3390/genes10050355

**Published:** 2019-05-09

**Authors:** Ghulam Qanmber, Faiza Ali, Lili Lu, Huijuan Mo, Shuya Ma, Zhi Wang, Zuoren Yang

**Affiliations:** 1 State Key Laboratory of Cotton Biology, Key Laboratory of Biological and Genetic Breeding of Cotton, Institute of Cotton Research, Chinese Academy of Agricultural Sciences, Anyang 455000, China; gqkhan12@gmail.com (G.Q.); faizabiochemist2017@gmail.com (F.A.); biolll@126.com (L.L.); mohuijuan86@163.com (H.M.); msymsy89@126.com (S.M.); 2 Zhengzhou Reseach Base, State Key Laboratory of Cotton Biology, Zhengzhou University, Zhengzhou 4550001, China

**Keywords:** *Gossypium hirsutum*, *GhHH3*, phylogenetic analysis, gene duplication, cis-elements, expression pattern, abiotic stress, phytohormonal stress

## Abstract

Histone acts as the core for nucleosomes and is a key protein component of chromatin. Among different histone variants, histone H3 (HH3) variants have been reported to play vital roles in plant development. However, biological information and evolutionary relationships of *HH3* genes in cotton remain to be elucidated. The current study identified 34 *HH3* genes in *Gossypium hirsutum*. Phylogenetic analysis classified *HH3* genes of 19 plant species into eight distinct clades. Sequence logos analysis among *Arabidopsis*, rice, and *G. hirsutum* amino acid residues showed higher conservation in amino acids. Using collinearity analysis, we identified 81 orthologous/paralogous gene pairs among the four genomes (A, D, At, and Dt) of cotton. Further, orthologous/paralogous and the Ka/Ks ratio demonstrated that cotton *HH3* genes experienced strong purifying selection pressure with restricted functional divergence resulting from segmental and whole genome duplication. Expression pattern analysis indicated that *GhHH3* genes were preferentially expressed in cotton ovule tissues. Additionally, *GhHH3* gene expression can be regulated by abiotic stresses (cold, heat, sodium chloride (NaCl), and polyethylene glycol (PEG)) and phytohormonal (brassinolide (BL), gibberellic acid (GA), indole-3-acetic acid (IAA), salicylic acid (SA), and methyl jasmonate (MeJA)) treatments, suggesting that *GhHH3* genes might play roles in abiotic and hormone stress resistance. Taken together, this work provides important information to decipher complete molecular and physiological functions of *HH3* genes in cotton.

## 1. Introduction

Histone is the major component of chromatin and histone genes have been widely studied in animals at the genome level. Histone genes in animals are classified according to whether they are replication independent or dependent, or on a tissue-specific basis. However, few studies have been conducted to elucidate the molecular functions of histone genes at the genomic level in plants [1]. Chromatin not only acts as DNA packaging in most eukaryotic cells, but also serves as a barrier in order to control accessibility of DNA. Histone has an essential role in chromatin structure modulation for various cellular processes such as DNA repair, replication, and transcription, as well as recombination. In chromatin, the nucleosome is the basic unit containing an octameric histone core and a DNA segment around that core [2]. Histone proteins are highly conserved among different plant species and are divided into histones H1, H2A, H2B, H3, and H4. Moreover, histone protein variants have been discovered based on different amino acids, which may vary from few amino acids to an entire protein segment [3,4,5]. 

During the last two decades, many studies have been conducted to explore post-transcriptional modifications involving histone that affect chromatin status including nucleosome stability and inter-nucleosomal contacts. It is now well established that the incorporation of histone variants results in chromatin formation with some particular features [6,7,8]. These histone variants are deposited in a DNA replication dependent manner. The deposition of a H3.1 histone variant may affect transcription of target genes [3,4,5]. In plants, different types of histone variants have been identified, including H3.1 and H3.3, on the basis of replication dependent and independent sub types. Unlike H3.1, histone variant H3.3 is incorporated into chromatin in a mostly replication-independent manner [9]. These histone variants differ from each other on the position of amino acids 31 and 87–90 [10,11]. 

Histone variant H3.3 genome-wide profiling in mammalian and *Drosophila* cells indicates specific incorporation into the body of active genes and regulatory elements, demonstrating the roles of H3 protein in transcription [12,13,14,15]. Another study indicated H3.3 enrichment in telomeres and peri-centric heterochromatin [16]. Moreover, several other studies demonstrated the particular functions of histone genes in genomic instability, sex chromosome inactivation, gene inactivation, and gene silencing [15,17,18]. Previously, the *HH3* gene *AtMGH3* was identified as a male-gamete-specific variant using a genome-wide approach in *Arabidopsis* [1]. Similarly, genome-wide analysis of HH3.1 and HH3.3 variants depicted similar genomic localization patterns with some unique features in *Arabidopsis* [4]. Another study showed that the presence of H3.3 in transcribed regions was strongly associated with transcriptional activity and H3.3 at promoters is often independent of transcription [3].

Cotton is an important natural fiber crop and a major contributor to the global textile industry. *HH3* family genes have been widely analyzed in *Arabidopsis* and rice, but there is no comprehensive analysis of *HH3* genes in cotton. Cotton genome sequencing during the last decade [19,20,21,22,23,24] has made it possible to analyze different cotton gene families with the help of genome-wide approaches. 

Here, we identified 34 *HH3* genes in *Gossypium hirsutum*. Phylogenetic analysis, sequence logo analysis for conserved amino acid residues, chromosomal distribution, gene duplication via collinearity analysis, Ka/Ks ratio, promoter cis-elements, gene structure, and domain architecture were predicted. Moreover, transcriptomic data analysis as well as expression pattern analysis of *GhHH3* genes in different cotton tissues was also estimated using qRT-PCR. Additionally, the expression patterns of *GhHH3* genes under various abiotic stresses and phytohormonal stimulus conditions were also determined to explore the functional roles of these genes in cotton. This work lays a foundation to elucidate the evolutionary and functional analysis of *GhHH3* genes in addition to exploring molecular and biological mechanisms to understand cotton biology. 

## 2. Materials and Methods 

### 2.1. Sequence Identification

To identify the *HH3* gene family members in different plant species, *Arabidopsis* HH3 protein sequences were used as query to retrieve *HH3* genes in *Gossypium arboreum* (ICR, version 1.0), *G. hirsutum* (NAU, version 1.1), *Gossypium raimondii* (JGI, version 2.0), *Brassica napus* (version 1.0), *Glycine max* (version 10), *Medicago truncatula* (version 10), *Populus trichocarpa* (version 2.0), *Solanum tuberosum* (version 10), *Theobroma cacao* (version 10), *Vitis vinifera* (version 10), *Oryza sativa* (version 10), *Zea mays* (version 10), *Sorghum bicolor* (version 10), *Ananas comosus* (version 3.0), *Chlamydomonas reinhardtii* (version 5.5), *Physcomitrella patens* (version 3.3), *Selaginella moellendorffii* (version 1.0), and *Pinus taeda* (version 1.0). The *Arabidopsis* database was downloaded from TAIR 10 (http://www.arabidopsis.org), while a database for *G. arboreum* was downloaded from ftp://bioinfo.ayit.edu.cn/downloads/. *G. hirsutum* and *G. raimondii* databases were downloaded from COTTONGEN (https://www.cottongen.org/). Databases for all other plant species were downloaded from Phytozome v11 (https://phytozome.jgi.doe.gov/pz/portal.html). To confirm these retrieved HH3 protein sequences, other bioinformatics approaches including Interproscan 63.0 [25] (http://www.ebi.ac.uk/InterProScan/) and SMART [26] (http://smart.embl-heidelberg.de/) were used. Further, biophysical properties such as number of amino acids (protein length), MW (molecular weight), pl (isoelectric point), and gravity values for *GhHH3* genes were calculated using the ExPASy ProtParam tool (http://us.expasy.org/tools/protparam.html). Moreover, subcellular localization of *GhHH3* genes was predicted using the online tool softberry (www.softberry.com). 

### 2.2. Phylogenetic and Conserved Sequence Analysis

For phylogenetic analysis, full length protein sequences of all observed species were aligned and two phylogenetic trees were generated in MEGA 7.0 [27] using the neighbor-joining (NJ) method. To determine the reliability of clades, the bootstrap method with 1000 replicates was used. Substitution was estimated using a Poisson model with default parameters. To generate sequence logos for conserved amino acid residues, multiple sequence alignment of *Arabidopsis*, rice, and *G. hirsutum* was carried out separately by Clustal X 2.0 (http://www.clustal.org/clustal2/) and sequence logos were generated by online software, WEBLOG [28]. 

### 2.3. Gene Structure, Domain Architecture, and Cis-Elements Analysis

*GhHH3* genes were first aligned by Clustal X 2.0 and then MEGA 7.0 to generate an NJ tree. The obtained bed-file was subjected to GSDS 2.0 [29] (http://gsds.cbi.pku.edu.cn/index.php). Domain architecture analysis was performed by subjecting full length protein sequences to MEME software [30] (http://meme-suite.org/tools/meme), as described previously [31]. For cis-element analysis of *GhHH3* gene promoter regions, sequence regions 2 kb upstream of the start codon were considered as proximal promoter regions and subjected to PlantCARE Database [32], and predicted cis-elements were classified according to their functional relevance, as described previously [33]. 

### 2.4. Chromosomal Localizations, Collinearity, and Ka/Ks Ratio Analysis

The chromosomal positions of *GhHH3* genes were first determined by cotton genome annotation file (ftp://ftp.bioinfo.wsu.edu/species), after which gff3-files were extracted. To draw their physical location on corresponding chromosomes, MapInspect software (https://mapinspect.software.informer.com/) was used. During collinearity, analysis orthologous/paralogous data were obtained by the previously described methods [34], and then a figure was generated using CIRCOS software [35]. Next, non-synonymous (Ka) and synonymous (Ks) divergence level ratios were calculated by aligning duplicated gene pair protein sequences in Clustal X 2.0, after which they were translated into complementary DNA (cDNA) sequences using the PAL2NAL program [36] (http://www.bork.embl.de/pal2nal/). Finally, Ka and Ks values were calculated with the help of the CODEML program using the PAML package [37]. 

### 2.5. Plant Material, Treatment, and qRT-PCR Analysis 

In this study, *G. hirsutum* variety CRI24 was used as genetic material to determine tissue specific expression patterns, as well as for abiotic and hormonal stresses. Specifically, pre-germinated cotton seeds were suspended in a container of liquid culture medium, as described previously [38]. For abiotic stress, four-week-old seedling at the 3–4 leaf stage were subjected to cold (4°C), heat (38°C), sodium chloride (NaCl) (300 nM), and polyethylene glycol (PEG) (20% mass fraction) for 1, 2, 4, and 6 hours. Similarly, seedlings at the same stage were subjected to five hormonal stresses including brassinolide (BL) (10 µM), gibberellic acid (GA) (100 µM), indole-3-acetic acid (IAA) (100 µM), salicylic acid (SA) (10 µM), and methyl jasmonate (MeJA) (10 µM) for 0.5, 1, 3, and 5 hours. After treatment, leaves were collected for RNA extraction and subsequent analysis. For tissue specific expression pattern analysis, 15 tissues including root, stem, leaf, flower, ovule (1, 3, 5, 7, 10, 15, and 20 days post-anthesis (DPA) ovule), and fiber (7, 10, 15, and 20 DPA fiber) were collected from cotton grown under field conditions with standard practices. Collected samples were immediately frozen in liquid nitrogen and stored at −80°C until used. 

RNA from collected tissues was extracted using an RNAprep Pure Plant Kit (TIANGEN, Beijing, China) and 1 µg of total RNA was reverse transcribed into cDNA with a PrimeScript® RT reagent kit (Takara, Dalian, China). For qPCR, Premix Ex TaqTM II (Takara) was used and PCR amplification was conducted on a LightCycler 480 (Roche Diagnostics GmbH, Mannheim, Germany). *GhUBQ7* (accession No. DQ116441) was used as an internal control. Each experiment was conducted in three independent biological repeats; primers used in this study are shown in Table S1. 

## 3. Results

### 3.1. Identification of HH3 Family Members

We identified a total of 257 HH3 proteins among 19 different plant species with a combination of methods. Of these, 14 members belonged to *Arabidopsis* (six H3.1, seven H3.3, and one centromeric variant); 13 each from *G. arboreum* (nine H3.1 and four H3.3 variants) and *G. raimondii* (eight H3.1 and five H3.3 variants); 34 from *G. hirsutum* (twenty-three H3.1, nine H3.3, and two centromeric variants); 17 from *G. max*; 21 from *M. truncatula*; 13 from *O. sativa*; 14 from *P. trichocarpa*; 15 from *S. bicolor*; 14 from *S. tuberosum*; 9 from *T. cacao*; 4 from *V. vinifera*; 12 from *B. napus*; 16 from *Z. mays*; 10 from *A. comosus*; 11 from *C. reinhardtii*; 10 from *P. patens*; six from *S. moellendorffii*; and 11 genes from *P. taeda*. We found that almost all selected plants have at least four *HH3* genes, with *G. hirsutum* having the highest number (34) of *HH3* genes and *V. vinifera* having only 4 genes, indicating that *HH3* genes were subjected to a large-scale expansion. (Table S2). These members of the *HH3* gene family were confirmed using different bioinformatics approaches including InterProscan 63.0 (http://www.ebi.ac.uk/interpro/), SMART (http://smart.embl-heidelberg.de/), and PROSITE (http://prosite.expasy.org/). As our main focus was *G. hirsutum*, we compared the genes from two *G. hirsutum* sequenced genomes (NAU and BJI) and found that all the genes were highly similar and the genes from NAU contained all genes of BJI. We used the genes from the NAU genome sequence database for our subsequent analysis. In our study, *HH3* genes in the AD cotton (*G. hirsutum*) genome were more than double the A (*G. arboreum*) and D genome (*G. raimondii*) cottons, which was consistent with tetraploid and diploid genomes and indicated the effects of polyploidy in AD genome cotton. 

Further basic information of *GhHH3* genes indicated the equal distribution on A and D chromosomes, as both had 17 genes. Their chromosomal location start and end points, strand, gene length, coding sequences (CDs), protein length, molecular weight (MW), isoelectric point (pl), gravity, and predicted cellular localization are provided in Table S3. *GhHH3-5* had the maximum coding region length (1134 bp) while *GhHH3-7* had only 297 bp of coding sequence. A similar pattern was observed for protein length and molecular weight of *GhHH3* family genes. The isoelectric points were 9.67 and 11.85 for *GhHH3-24* and *GhHH3-7*, respectively. Moreover, gravity was −0.607 and −0.047 for *GhHH3-15* and *GhHH3-24*, respectively. However, predicted subcellular localization results indicted the cellular localization of all *GhHH3* genes in the nucleus (Table S3). 

### 3.2. Phylogenetic Analysis of HH3 Genes

**Figure 1:**
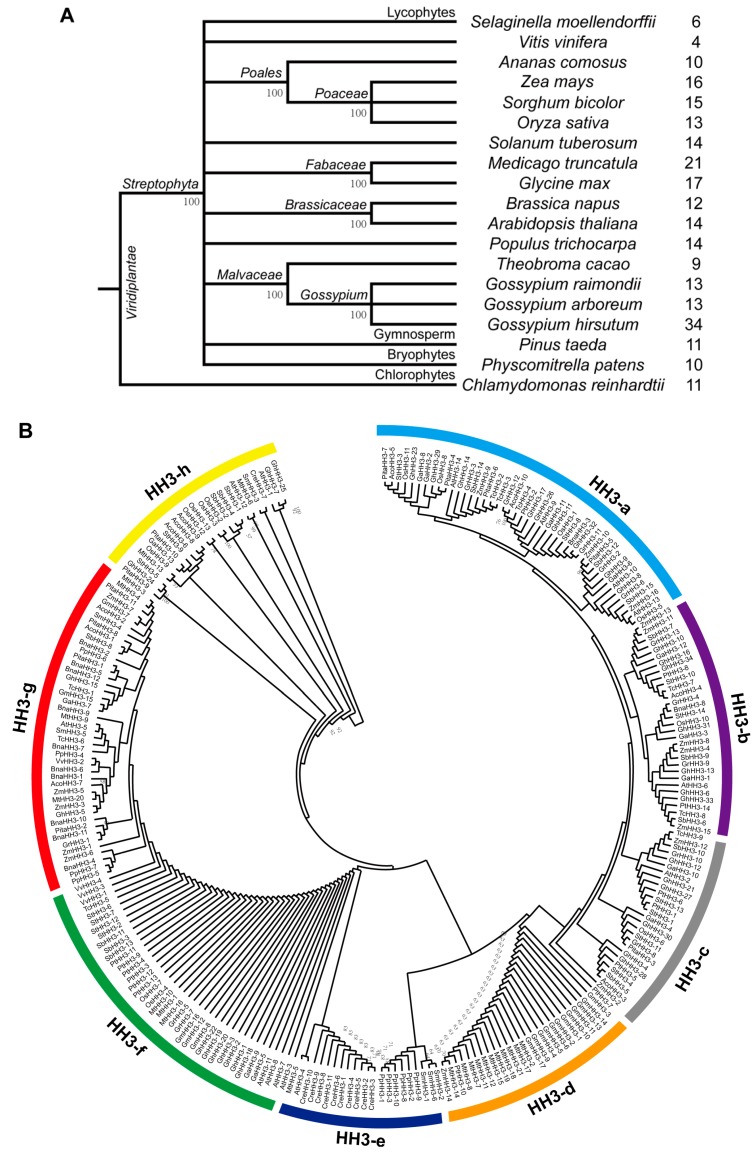
Phylogenetic tree of histone H3 (*HH3*) genes from 19 plant species. (**A**) The phylostratum analysis of the *HH3* gene family. (**B**) Phylogenetic tree divided all 257 *HH3* genes into eight clades from HH3-a to HH3-h. Bootstrap values were also mentioned near the node of each branch.

The phylostratum analysis of the *HH3* gene family identified the earliest plant lineage as *HH3* genes were present in *C. reinhardtii* (chlorophyte), an early plant lineage (Figure 1A). *HH3* genes were present in *A. comosus* (angiosperm), *P. taeda* (gymnosperm), *P. patens* (bryophytes), *S. moellendorffii* (lycophytes), dicotyledons (*A. thaliana, G. arboreum, G. hirsutum, G. raimondii, B. napus, G. max, M. truncatula, P. trichocarpa, S. tuberosum, T. cacao*, and *V. vinifera*), and monocotyledons (*O. sativa, Z. mays*, and *S. bicolor*). These results indicated that *HH3* genes originated from early land plants’ phylostratum, and potential orthologous genes of *HH3* are present throughout the plant kingdom. Next, we built an NJ tree to estimate the deeper relationship of *HH3* genes of 19 plant species including dicotyledons (*A. thaliana, G. arboreum, G. hirsutum, G. raimondii, B. napus, G. max, M. truncatula, P. trichocarpa, S. tuberosum, T. cacao*, and *V. vinifera*), monocotyledons (*O. sativa, Z. mays*, and *S. bicolor*), *A. comosus* (angiosperm), *P. taeda* (gymnosperm), *C. reinhardtii* (chlorophyte), *P. patens* (bryophytes), and *S. moellendorffii* (lycophytes). The prefixes At, Ga, Gh, Gr, Bna, Gm, Mt, Pt, St, Tc, Vv, Os, Zm, Sb, Aco, Cre, Pp, Sm, and Pita were used before the names of *HH3* genes from *A. thaliana, G. arboreum, G. hirsutum G. raimondii, B. napus, G. max, M. truncatula, P. trichocarpa, S. tuberosum, T. cacao*, *V. vinifera*, *O. sativa, Z. mays*, *S. bicolor*, *A. comosus*, *C. reinhardtii*, *P. patens*, *S. moellendorffii*, and *P. taeda*, respectively. The NJ tree showed that all 257 *HH3* genes from 19 different plant species were naturally classified into eight clades, a–h (Figure 1B). 

In this study, clade HH3-a contained the most *HH3* genes (45 genes) followed by clade HH3-g (43 genes), clade HH3-f (40 genes), clade HH3-b (31 genes), clade HH3-c (27 genes), clade HH3-d (25 genes), clade HH3-h (25 genes), and clade HH3-e (21 genes). HH3-a and HH3-c clades contain genes from *A. comosus* (angiosperm), *P. taeda* (gymnosperm), dicots and monocots, except for *S. moellendorffii* (lycophytes), *C. reinhardtii* (chlorophyte), and *P. patens* (bryophytes). HH3-b lacks the genes from *S. moellendorffii*, *C. reinhardtii*, *P. patens*, and *P. taeda*, while *HH3* genes from *G. hirsutum*, *A. comosus*, *S. moellendorffii*, *C. reinhardtii*, *P. patens*, and *P. taeda* were absent in HH3-d clade and have the majority of *HH3* genes from *G. max* and *M. truncatula*. Similarly, HH3-e clade contains genes from *S. moellendorffii*, *C. reinhardtii*, and *P. patens*, except for one gene from *A. thaliana* and *M. truncatula*. *HH3* genes from *A. comosus*, *S. moellendorffii*, *C. reinhardtii*, *P. patens*, and *P. taeda* were absent in HH3-f clade. However, *HH3* genes from *C. reinhardtii* and *P. patens* were absent in HH3-g and HH3-h clades respectively. Among the eight clades, *HH3* genes from *G. hirsutum* were distributed in six clades (except HH3-d and HH3-e) of the phylogenetic tree. All clades (except HH3-e) clustered genes from both monocot and dicot plant species, indicating that the *HH3* gene family existed before the separation of mono- and dicotyledons. Although genes from dicots or monocots were closely clustered to each other, *G. hirsutum* experienced a significant increase in genes as their members were more than double all other observed species, except for *M. truncatula.* Gene enlargement in *G. hirsutum* can be observed in our phylogenetic analysis, as its nine pairs of genes were closely clustered to each other. Further, paralogous gene pairs derived from the same node were observed in almost all observed species (except *P. taeda*), even *V. vinifera*, although it only had four *HH3* members (Figure 1B). These paralogous pairs were the result of duplication and indicated that *HH3* genes experienced a duplication event in their evolution that contributed toward gene family expansion. However, this duplication was uneven in all clades and different species. 

### 3.3. Conserved Amino Acid Residues Analysis

**Figure 2:**
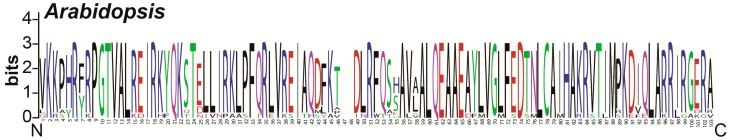

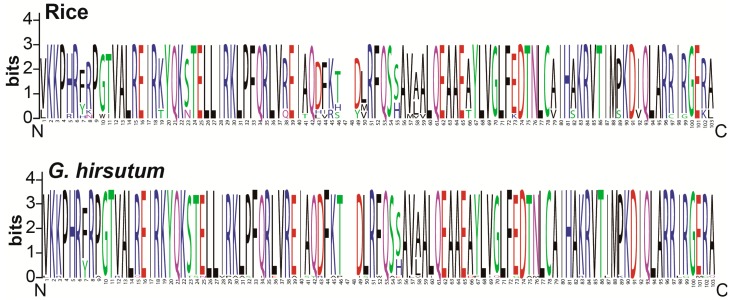
Sequence logos of conserved amino acid residues generated for three plant species including *Arabidopsis* (first), rice (second), and *Gossypium hirsutum* (third) exhibited highly conserved sequence logos during the evolution of dicots and monocots plant species.

To find homologous domain sequences and to investigate the conservation of each amino acid residue in GhHH3 domains, a multiple sequence alignment was performed to construct sequence logos in *Arabidopsis*, rice, and *G. hirsutum*. The results indicated that the amino acid residue distribution was highly similar at most of the loci among all three observed plant species. For instance, some amino acid residues including V [1], K [2], K [3], P [4], H [5], R [6], P [9], V [12], A [13], L [14], R [15], E [16], R [18], Q [21], K [22], T [24], E [25], L [26], L [27], R [35], L [36], V [37], I [39], R [40], A [41], A [42], L [43], Q [44], E [45], A [46], A [47], E [48], and so on were found to be highly conserved. No composition bias of any specific conserved amino acid residue was observed in any region, suggesting a highly conserved distribution pattern irrespective of the N or C terminal end of their domain in all observed species (Figure 2). 

### 3.4. Chromosomal Locations, Duplication, and Collinearity Analysis of GhHH3 Members

Next, we mapped the *GhHH3* genes onto their corresponding chromosomes with the GFF3 file. The *GhHH3* genes were found to be equally distributed on At and Dt sub-genome chromosomes of AD cotton. All 34 *GhHH3* genes were allotted to eight At sub-genomes as well as to eight Dt sub-genome chromosomes (Figure S1). In total, 17 *GhHH3* genes were allotted to the At sub-genome and to the Dt sub-genome chromosomes, indicating the equal distribution of *GhHH3* genes in both sub-genomes. A maximum number of genes (four genes) was allotted to A10 and its corresponding D10 chromosome. However, D02, A06, D06, A07, and A11 contained only one *GhHH3* gene. A previous report indicated that translocation occurs between the A02 and A03 chromosome [22], which might be the major cause of gene distribution on homologous chromosomes. In contrast, no genes were mapped on A01, D01, A03, A04, D04, A09, D09, D11, A12, and D12 chromosomes. The equal number of genes mapped on chromosomes of At and Dt sub-genomes indicated that some genes might be lost during the process of evolution or that incomplete genome sequencing led to the identification of fewer genes than their actual numbers. 

**Figure 3:**
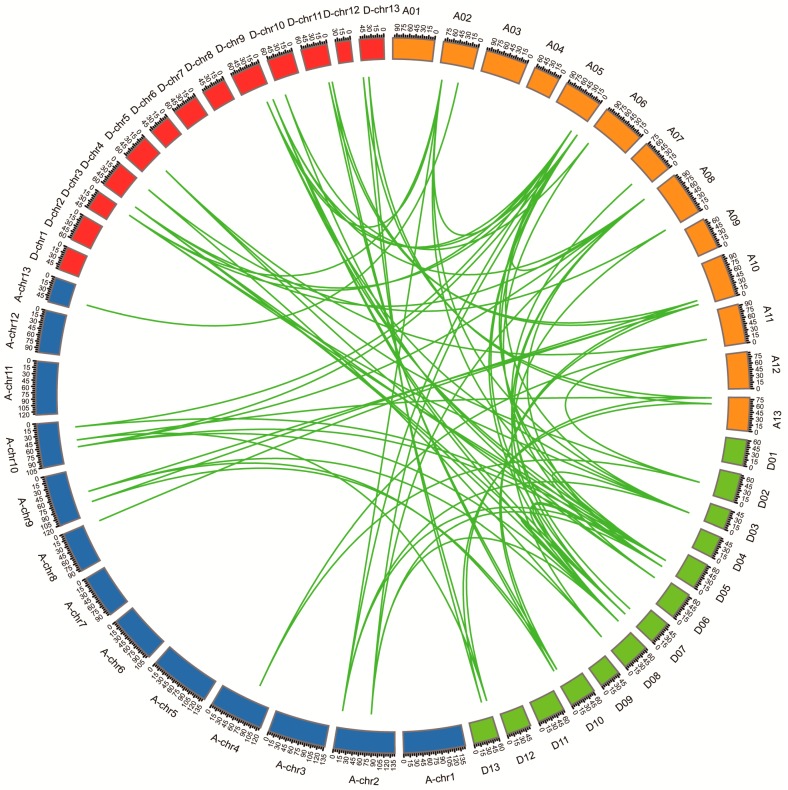
Gene duplication among *HH3* genes in cotton including *G. arboreum* (A-genome), *G. hirsutum* (At and Dt sub-genome), and *G. raimondii* (D-genome). Red lines linking two different genes represent the orthologous pairs diverged from the same ancestor. A01–A13 (mustard colored blocks) represent the chromosomes of At sub-genome and D01–D13 (light green colored blocks) exhibit the chromosomes of Dt sub-genome. Similarly, A-chr1-A-chr13 (blue colored blocks) and D-chr1-D-chr13 (red colored blocks) demonstrate *G. arboreum* and *G. raimondii* chromosomes, respectively.

As an allotetraploid, *G. hirsutum* serves as a model to study the effect of naturally occurring polyploidy [24]. In order to study the locus relationship between the orthologous of At and Dt sub-genomes, collinearity analysis was performed. The analysis showed that most *GhHH3* gene loci were highly conserved between the At and Dt sub-genomes (Figure 3 and Table S4). *G. hirsutum* is derived as the result of hybridization of two diploid cotton species resembling *G. arboreum* and *G. raimondii* and subsequent doubling of chromosomes [39,49]. In our study, we observed that each gene of the At or Dt sub-genomes had orthologous on A or D genomes and similarly the genes of any one of these four genomes (A, D, At, and Dt) had orthologous on any of the other three genomes. These findings indicated that cotton *HH3* genes were not subjected to genomic rearrangements during the event leading to polyploidy. Collinearity analysis indicated that two paralogous gene pairs were found within the At sub-genome (Figure 3 and Table S4). Overall, a total of 81 orthologous/paralogous gene pairs were identified among four genomes (A, D, At, and Dt). Coupled with these findings, we deduced that orthologous/paralogous gene pairs were derived as a result of a segmental or whole genome duplication (WGD) event before polyploidy occurred.

Over evolutionary time, the duplicated genes experienced functional divergence including non-functionalization (lack of original functions), sub-functionalization (partition of original functions), and neo-functionalization (acquiring new functions) [50,51]. To check whether Darwinian positive selection pressure is also related to the divergence of *GhHH3* family genes after duplication, we calculated the Ka/Ks (non-synonymous/synonymous) ratio of all identified orthologous/paralogous gene pairs. It has been established that Ka/Ks = 1.0 represents pseudogenes as a result of neutral selection, Ka/Ks < 1.0 demonstrates the tendency of duplicated genes for purifying selection, while ratio of Ka/Ks >1.0 exhibits positive selection of accelerated evolution. We found that the Ka/Ks ratio for 70 gene pairs were < 0.5, while that for eight gene pairs was between 0.5 and 1.0, and three exhibited a Ka/Ks ratio > 1.0 (Table S4), indicating that these three gene pairs experienced rapid evolution following duplication. As most Ka/Ks ratio were less than 1.0, we speculated that cotton *HH3* genes were subjected to strong purifying selection pressure with restricted functional divergence as the result of segmental and whole genome duplication (WGD). 

### 3.5. Promoter Cis-Element, Gene Structure, and Domain Architecture of the GhHH3 Gene Family 

To investigate transcriptional regulation as well as the potential functions of *GhHH3* genes, we predicted cis-elements in their promoter region and categorized them according to their related roles in plant growth and development, light, and stress responses. We found that the promoters of *GhHH3s* contain various cis-elements related to growth, development, light, and stress responses (Table S5). Most of the gene promoter regions contained various elements for plant growth and development including Skn-1, 3-AF1 binding site, CCGTCC-box, GCN4_motif, CAT-box, Sp1, circadian, dOCT, O2-site, and as-2-box. Similarly, light responsive cis-elements such as ACE, AE-box, ATCT-motif, Box 4, Box I, GA-motif, GAG-motif, GATA-motif, GT1-motif, MRE, and I-box were also detected in the promoters of various *GhHH3* genes. Further, ARE, Box-W1, CGTCA-motif, EIRE, ERE, GARE-motif, HSE, LTR, P-box, TC-rich repeats, TCA-element, TGA-element, TGACG-motif, and W box were also found in many gene promoter regions, depicting their relatedness to various stress responses. 

It has been reported that gene structure is associated with the evolution of different plant species [52], so gene structure analysis was used along with phylogenetic relationships to elucidate the evolutionary relationship among *GhHH3* genes. An NJ tree was generated and with exon/introns and different motifs comprising the *GhHH3* gene family (Figure S2A,B). All members of the *GhHH3* gene family lack introns and have only one exon (Figure S2A). Moreover, all *GhHH3* genes from At and Dt sub-genomes displayed a significant conserved motif pattern, as all the genes have the same motifs, except for *GhHH3-24*, *GhHH3-5*, *GhHH3-7*, and *GhHH3-25* (Figure S2B). Overall, all genes exhibited a highly conserved pattern of gene structure and motif throughout At and Dt sub-genomes. 

### 3.6. Expression Profiles of GhHH3 Members in Various Tissues and Developmental Stages

**Figure 4:**
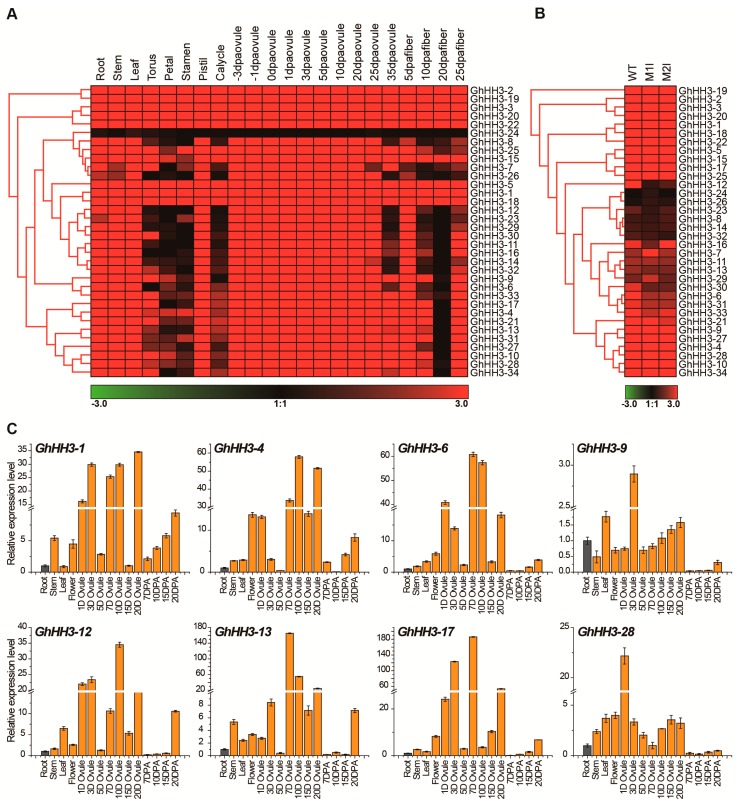
Tissue specific expression patterns analysis of *GhHH3* genes in different tissues of the plant. (**A**) Heat map generated for the expression of 34 *GhHH3* genes in 22 different tissues. Data obtained from publically available transcriptomic data and the color bar (down) indicate the values of expression level. (**B**) Heat map of all *GhHH3* relative expressions in two fuzzless/lintless mutants (*M1l and M2l*) as compared with wild type (WT) plants. Data were extracted from published RNA-seq data and the color bar (down) indicates the expression level. (**C**) Relative expression level of eight selected *GhHH3* genes in different tissue estimated by qRT-PCR analysis. Error bars indicate the standard deviations (SD) of three independent biological repeats.

Gene expression predicts the biological functions of a gene, so we inspected the expression pattern of *GhHH3* genes in different cotton tissues. First, we analyzed publicly available transcriptomic data downloaded from the National Center for Biotechnology Information (NCBI) (https://www.ncbi.nlm.nih.gov/pmc/articles/PMC4482290/) [53] and a heat map of all 34 *GhHH3* genes for 22 different tissues was created (Figure 4A). We observed that all *GhHH3* genes (except *GhHH3-24*) were widely expressed in vegetative (root, stem, and leaf) and reproductive (torus; petal; stamen; pistil; calycle; −3, −1, 0, 1, 3, 5, 10, 20, 25, and 35 DPA ovules) tissues, and fiber (5, 10, 20, and 25 DPA), demonstrating that *GhHH3* genes play various biological functions. Some *GhHH3* genes were not expressed in torus, petal, and stamen or at different fiber developmental stages. All genes expressing similar expression patterns were found closely clustered to each other. Further, we investigated the expression levels of *GhHH3* genes in RNA-seq data of two fuzzless/lintless mutants (*M1l* and *M2l*) [54,55]. The results of the heat map indicated that in the *M1l* mutant, 11 *GhHH3* genes were upregulated, while only 3 genes were downregulated. Similarly, 3 genes were found to be upregulated, while 10 genes were downregulated in the *M2l* mutant compared with wild type (WT). However, the *GhHH3-24* expression level was not affected in either mutant. Overall, 2 genes were upregulated, and 16 genes were downregulated commonly in both observed mutants (Figure 4B and Table S6).

Fiber is the most important yield trait of cotton crop, so we selected 8 *GhHH3* genes from 16 downregulated genes in both *M1l* and *M2l* mutants having significant differences. *GhHH3-19*, which has the common name *GhHis3*, was not highly expressed in the observed mutants, so it was used as a reference gene (internal control) for gene expression during qRT-PCR analysis in *G. hirsutum* [56]. The eight selected *GhHH3* genes were further used to estimate tissue specific expression levels by qRT-PCR in root, stem, leaf, flower, ovule (1, 3, 5, 7, 10, 15, and 20 DPA ovule), and fiber (7, 10, 15, and 20 DPA fiber) to validate the previous findings (Figure 4C). The results indicated that all eight observed genes exhibited ubiquitous expression patterns in all observed tissues. Consistent with transcriptomic data analysis, all genes except *GhHH3-1* had downregulated expression in different fiber stages, except at 20 DPA. Moreover, all observed genes had maximum expression in different stages of ovule (except for some stages at only some stages), similar to the transcriptomic data. This result indicated the validity of our findings and demonstrated that *GhHH3* genes might play functional roles in different stages of ovule development, as all genes had conserved expression in ovule development. From here, we may speculate that *GhHH3* genes were preferentially expressed in different stages of ovule development, elucidating their conserved role with limited functional divergence during evolution. 

### 3.7. Responses of GhHH3 Members Under Various Abiotic Stresses and Phytohormonal Treatments

**Figure 5:**
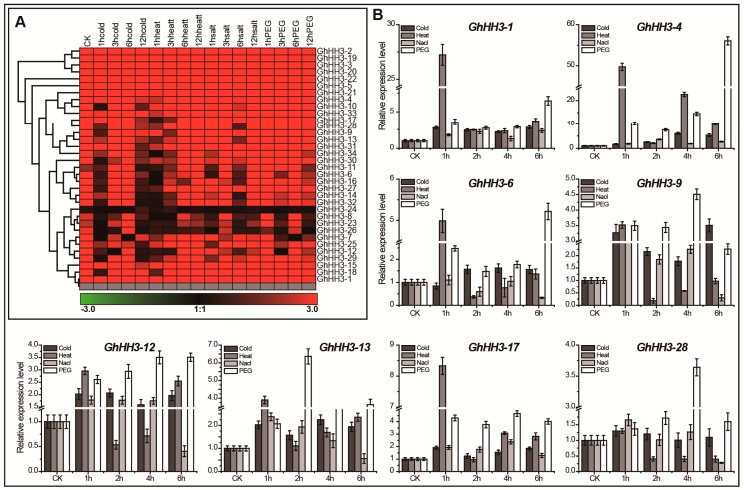
Expression patterns analysis of *GhHH3* genes under the treatment of four different abiotic stresses (cold, heat, sodium chloride (NaCl), and polyethylene glycol (PEG)). (**A**) Heat map of all *GhHH3* gene family members generated using publically available transcriptomic data. Color bar (down) indicated the response of that gene under specific treatment. (**B**) Expression pattern of eight *GhHH3* genes in cotton seedlings under same abiotic stresses estimated by qRT-PCR analysis. The error bars exhibit standard deviations (SD) of three independent biological repeats.

Cotton experiences various abiotic and hormonal stresses during its growth and development. Therefore, a comprehensive analysis of *GhHH3* gene expression under various abiotic stresses including cold, heat, salt, and PEG was performed in this study. First, we estimated the expression level of all 34 *GhHH3* genes using available transcriptomic data [22] and a heat map was generated. Genes depicting a similar response to different stresses were clustered together (Figure 5A). All genes showed widely variable responses and were upregulated (except *GhHH3-24*) under all observed stresses. However, the responses of some genes (including *GhHH3-8*, *GhHH3-23*, and *GhHH3-26*) were poor, but still upregulated at various stages. Further, the responses of eight selected genes under cold, heat, NaCl, and PEG were observed at different time points after treatment via qRT-PCR analysis (Figure 5B). 

All genes had an upregulated response many times higher than that of the control at different time points for each treatment (except at some time points for different treatments). Additionally, *GhHH3-1*, *GhHH3-4*, and *GhHH3-17* were fully upregulated at each time point for every stress stimulus. Moreover, *GhHH3-9* and *GhHH3-12* had downregulated expression only on heat exposure after 2, 4, and 6 h of treatment. Furthermore, *GhHH3-13* was only downregulated at 6 h after NaCl treatment only; otherwise, it was upregulated at all time points after every stress stimulus. Together with these findings, *GhHH3* genes exhibited obvious resistance against observed abiotic stresses, as their expression can be regulated by multiple stresses, suggesting that *GhHH3* genes are possible candidates for breeding stress resistant cotton. 

**Figure 6:**
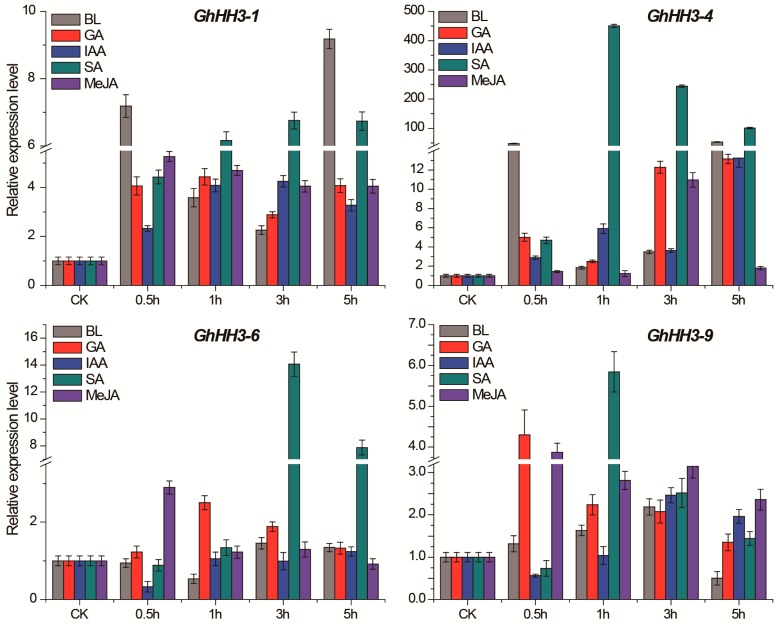

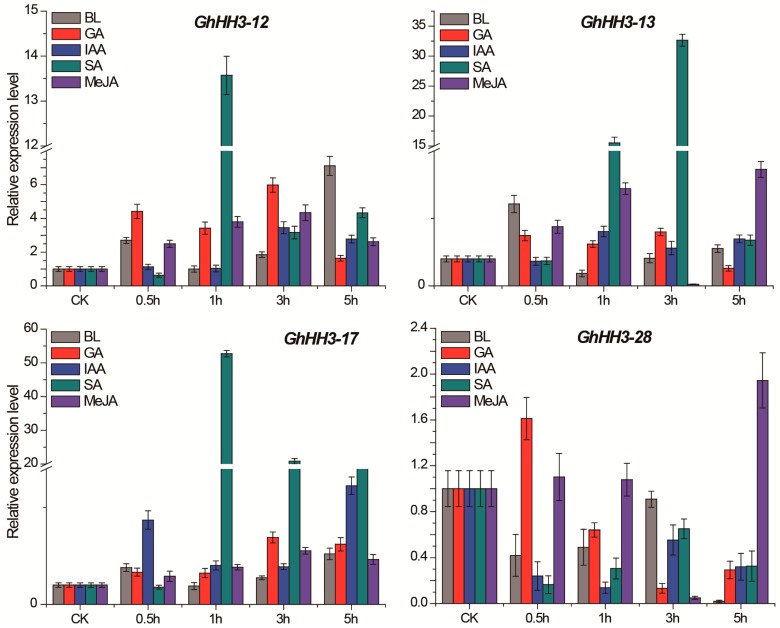
Expression analysis of selected *GhHH3* genes in cotton seedlings under exposure to five different phytohormonal stress stimuli including brassinolide (BL), gibberellic acid (GA), indole-3-acetic acid (IAA), salicylic acid (SA), and methyl jasmonate (MeJA). Error bars indicated standard deviations (SD) among three independent biological experiments.

To explore the functional and physiological relevance of *GhHH3* genes, we analyzed the expression pattern of eight selected genes under exposure to five phytohormonal stress stimuli including BL, GA, IAA, SA, and MeJA after 0.5, 1, 3, and 5 h of treatment (Figure 6). All *GhHH3* genes were found to be regulated by different phytohormones. Seven out of eight observed genes (except *GhHH3-28*) were highly upregulated on each phytohormonal exposure, except for slight downregulation at some points for specific hormones. Further analysis demonstrated that *GhHH3-1*, *GhHH3-4*, and *GhHH3*-17 were highly and significantly upregulated at different time points with different hormones. Only *GhHH3-28* was downregulated for most time points, except at a few including 0.5 h after GA and 0.5, 1, and 5 h after MeJA treatment. All genes except *GhHH3-28* were highly upregulated at 1 and 3 h after SA treatment. Moreover, expression of all genes (including some time points in the case of *GhHH3-28*) was positively regulated at all time points after MeJA treatment. In conclusion, *GhHH3* genes might play vital roles in phytohormonal cotton biology, as depicted by their regulation via various hormonal stress stimuli, and could prove an important resource for cotton biology improvement and subsequent studies. 

## 4. Discussion

Extensive studies have been conducted to explore the roles and biological functions of histone H3 genes, as well as histone modification in various plant species [1,3,5,15,16,40,41,42,43,57,58,59,60,61,62,63,64]. The analysis of the *HH3* gene family has been employed in *Arabidopsis* and rice in previous reports [1,2]. However, no systematic analysis of *HH3* genes in cotton has been performed to date. In the current study, we performed a comprehensive analysis of *HH3* genes in *G. arboreum, G. hirsutum*, and *G. raimondii*, but mainly focused on *G. hirsutum* to explore the roles of the *HH3* gene family in cotton that might help to lay a foundation for their future study. 

### 4.1. Cotton HH3s Have Been Highly Conserved During Evolution

In the current study, we identified 257 *HH3* genes in 19 different plant species including dicotyledons (*A. thaliana*, *G. arboreum*, *G. hirsutum*, *G. raimondii*, *B. napus*, *G. max*, *M. truncatula*, *P. trichocarpa*, *S. tuberosum*, *T. cacao*, and *V. vinifera*), monocotyledons (*O. sativa*, *Z. mays*, and *S. bicolor*), *A. comosus* (angiosperm), *P. taeda* (gymnosperm), *C. reinhardtii* (chlorophyte), *P. patens* (bryophytes), and *S. moellendorffii* (lycophytes). Previously, 15 and 16 *HH3* genes were reported in *Arabidopsis* and rice genomes, respectively, but evolutionary analyses of these genes have been conducted separately [1,2]. The phylostratum analysis of the *HH3* gene family identified the earliest plant lineage as *HH3* genes were present in *C. reinhardtii* (chlorophyte), indicating that *HH3* genes originated from early land plants’ phylostratum, and potential orthologous genes of *HH3* are present throughout the plant kingdom. Phylogenetic analysis indicated that all *HH3* genes can be naturally classified into eight major clades. Phylogenetic tree showed that HH3-g and HH3-h clades are ancient groups having *HH*3 genes from all selected plant species except *C. reinhardtii* and *P. patens*, respectively, while the HH3-f clade could be more advance than others, lacking *HH3* genes from *A. comosus*, *S. moellendorffii*, *P. taeda*, *C. reinhardtii*, and *P. patens*. The presence of *HH3* genes in each selected plant species, with 34 *HH3* genes from *G. hirsutum* and *V. vinifera* having only four genes, demonstrated that *HH3* genes are evolutionarily conserved and experienced a large-scale expansion in plants. Further, in seven clades, the genes from monocots and dicots were evenly distributed, suggesting that the *HH3* gene family is an ancient gene family in plants. Previously, the phylogenetic analysis of *HH3* genes has been conducted in *Arabidopsis* and rice. There have been no phylogenetic analyses of *HH3* among dicotyledons, monocotyledons, angiosperm, gymnosperm, chlorophyte, bryophytes, and lycophytes. 

In this study, multiple sequence alignment used to generate sequence logos of conserved amino acid residues for monocots (rice) and dicots (*Arabidopsis* and *G. hirsutum*) indicated no composition bias of any specific conserved amino acid residue. In addition, all three observed species sequence logos were highly conserved, irrespective of N or C terminus. Previous reports indicated that histone proteins are highly conserved even in different plant species, although a series of variants have been discovered on the basis of amino acid differences in their sequences. These differences might range from a few amino acids to a large portion of a protein. Histone variant H3 was found to be positively associated with gene transcription. Previous genome-wide analysis of *HH3* enrichment was observed toward promoters and transcription termination sites [3,4,5]. In the current study, amino acid residues in sequence logo analysis such as V, K, K, P, H, R, P, V, A, L, R, E, R, Q, K, T, E, L, L, R, L, V, I, R, A, A, L, Q, E, A, A, E, and so on were highly conserved. 

The molecular weight, isoelectric point, and predicted subcellular localization of these genes were almost the same. The promoter sequences of all *GhHH3* genes had almost the same distribution of cis-elements related to growth and development, as well as light and stress responses. Various studies have elucidated the high impact of light on plant growth and differentiation [44]. Cis-elements such as heat stress response element [45], abscisic acid (ABA) responsive elements [46], and dehydration-response elements [47] have been identified. Further, cis-elements for cold, TCA, and CGTCA elements regulate gene expression following exposure to MeJA and SA stress, respectively [48,65]. Moreover, TATC, P-box, and ethylene responsive elements were also observed. Additionally, presence of W-box conferred responses to ABA and drought stress [66]. The majority of *GhHH3* genes containing these elements with typical features demonstrated the predicted functions in growth, development, abiotic, and hormonal stresses. 

The distribution of *GhHH3* genes on corresponding chromosomes illustrated the equal distribution on At- and Dt- sub genome chromosomes, as both contain 17 genes. Gene structure and protein motif distribution of all *GhHH3* genes, except for a few, were highly similar, demonstrating that *GhHH3* genes were highly conserved. It has been reported based on gene structure that introns played essential roles during the evolution of various plant species [52]. The established phenomenon is that there were more introns during the early expansion phase, followed by a subsequent decrease over time [67]. These findings showed that more advanced species had fewer introns in their genomes [68]. More or larger introns lead toward the generation of new functions. 

Tandem duplications result in an increase in introns and the subsequent generation of some new genes [69]; however, we did not find evidence of tandem duplications in the current study. As *GhHH3* genes did not experience tandem duplication (but only underwent segmental and whole genome duplication), *GhHH3* genes have no introns and a lack of new genes and functional divergence. These findings were consistent with some previous reports. The lack of introns suggests that the *GhHH3* gene family is an advanced gene family where introns were lost over evolutionary time, and that these genes have evolutionarily conserved functions in cotton growth and development. 

### 4.2. Cotton HH3 Gene Enlargement During Evolutionary Processes

As an allotetraploid, cotton is an ideal material to investigate the effects of polyploidy [39]. The cotton A-genome (native to Africa and resembles *G. arboreum* and *G. herbaceum*) and the D-genome (native to Mexico and resembles *G. raimondii*) diverged about 5–10 mya (million years ago). Hybridization between the A- and D-genomes resulted in the doubling of chromosomes and eventually the generation of nascent AtDt (allotetraploid upland cotton) genomes occurred [49]. 

Here, a total of 34 *GhHH3* genes were identified, a higher number of *HH3* genes than any other observed species. Polyploidy is an important feature of upland cotton, which doubled the number of *GhHH3* gene members as a result of segmental or whole genome duplication (WGD). A previous study indicated that a polyploidy event occurred during the evolution of flowering plants that helped them to adapt to new environmental conditions [70]. The number of *HH3* genes in upland cotton increased significantly, as evidenced by a comparison with A- (*G. arboreum*) and D-genome (*G. raimondii*) cottons. However, gene loss always happens after hybridization during the phase of enhanced arrangement in the genomic sequences and chromosome doubling [71]. Compared with paleopolyploid maize as well as *Brassica*, the cotton genome exhibited minor changes [72,73]. 

Polyploidy mainly contributes toward duplication, and segmental as well as whole genome duplication are the main reasons for increased *GhHH3* family members, as both duplication events occur frequently in plants [74]. Plant species such as *Arabidopsis* experienced WGD twice in the *Brassicaceae* lineage [75]. Similarly, cotton and cacao have the same ancestor and faced ancient duplication about 18–58 mya. Eventually, there was another nascent duplication event in cotton [23].

In our study, we did not find evidence of tandem duplication even though it is the main contributor to gene family expansion as the result of unequal crossing over, transposons insertion, chromosomal anomalies, as well as other reverse transcriptase-mediated processes. Duplication results in new genes that are redundant with old ones and this redundancy is considered a driving force of evolution [76]. A previous study indicated that gene duplication causes redundancy of the *HH3* genes in *Arabidopsis* [1]. In the current study, we identified only two pairs of segmental duplications, while WGD contributed more in *GhHH3* gene family enlargement. 

Previously, sesame heat shock protein gene family expansion was found to be the result of segmental duplication [77]. Similarly, soybean *WRKY*, cotton *GRAS*, *WOX*, *YABBY*, *MIKC*-Type *MADS*-Box, and *RING-H2* finger E3 ligase gene families also expanded as the result of segmental as well as whole genome duplication [34,77,78,79,80,81,82,83]. In light of the above findings and previous reports, we concluded that A-genome *HH3* genes and At sub-genome *GhHH3* genes had the same common ancestor, and the D-genome and Dt sub-genome *HH3* genes also had the same common ancestor. Further, the phylogenetic and collinearity analysis strengthened our speculation that *GhHH3* genes were highly conserved with limited functional divergence during evolution. 

### 4.3. Functional Diversifications of GhHH3 Family Members 

Several studies have been conducted to explore the biological roles of *HH3* genes in many plant species including *Arabidopsis* and rice [3,4,5,64]. To our knowledge, there has been no systematic study to explore the molecular functions of *HH3* genes in cotton. Tissue-specific expression levels of eight selected *GhHH3* genes indicated that *GhHH3* genes were highly expressed and played a positive role at various observed stages of ovule development. Precisely, all eight genes including *GhHH3-1*, *GhHH3-4*, *GhHH3-6*, *GhHH3-9*, *GhHH3-12*, *GhHH3-13*, *GhHH3-17*, and *GhHH3-28* were significantly positively expressed in different stages of ovule development, suggesting their roles in the ovule development process. These results were consistent with previously published transcriptomic data. However, all observed genes except *GhHH3-1* did not have a significant contribution toward cotton fiber development, similar to the results of transcriptomic data. 

Further, in four abiotic stress conditions (cold, heat, NaCl, and PEG), all genes were positively regulated many times higher than the control at different time points. However, two genes, *GhHH3-9* and *GhHH3-12*, had downregulated expression on heat exposure after 2, 4, and 6 h of treatment; in addition, *GhHH3-13* was downregulated at 6 h after NaCl treatment. These findings were consistent with published transcriptomic data. 

Further, the analysis of *GhHH3* genes for five different hormonal treatments including BL, GA, IAA, SA, and MeJA at different time points indicated that expression of all genes was regulated at different time points of observations. Particularly, all genes except for *GhHH3-28* were upregulated following exposure to SA and MeJA, depicting their particular roles under the exposure of these phytohormonal treatments. Coupled with these results, we found that *GhHH3* genes preferentially expressed during different stages of ovule development. In addition, *GhHH3* genes expression can be regulated by abiotic and hormonal stress stimuli responses, suggesting that *GhHH3* genes are possible candidates for breeding abiotic and hormonal stresses in cotton. 

## 5. Conclusions

Previous studies deliberately illustrated the biological role of HH3 proteins in different plant species. In the current study, we systematically identified 257 *HH3* genes in 19 different plant species, including 34 *HH3* genes in upland cotton *G. hirsutum*. Phylogenetic analysis classified these 257 *HH3* genes into eight well categorized clades with polyploidy and duplication effects. All *GhHH3* genes lacked introns and had highly conserved protein motif distributions. Conserved amino acid sequence logos of *Arabidopsis*, rice, and *G. hirsutum* indicated that *HH3* genes were highly conserved during evolution. Cis-elements with particular features in the promoter regions of *GhHH3* genes indicated their functional relatedness to growth, development, and stress responses. At- and Dt-sub genomes had an equal distribution of 34 *GhHH3* genes on the chromosomes. Duplication and collinearity analysis of *GhHH3* genes indicated that cotton *HH3* genes experienced segmental and whole genome duplication over evolutionary time. Further, duplicated gene pairs had limited functional divergence and were highly conserved demonstrating that A-genome *HH3* genes and At sub-genome *GhHH3* genes had the same common ancestor, and D-genome and Dt sub-genome *HH3* genes also had the same common ancestor. In addition, expression analysis showed that *GhHH3* genes preferentially expressed in ovule development along with the fact that these genes expression can be regulated by abiotic and hormonal stresses and might prove a possible genetic material in cotton breeding for abiotic and hormonal stress conditions.

## Supplementary Materials

The following are available online at https://www.mdpi.com/2073-4425/10/5/355/s1. Figure S1: Chromosomal distribution of *GhHH3* genes on different chromosomes of *G. hirsutum*. A02 to A13 and D02 to D13 represent At and Dt sub-genomes *G. hirsutum*, respectively; Figure S2: Gene structure and domain architecture of *GhHH3* genes along with phylogenetic tree constructed by NJ method. (a) Gene structure of all *GhHH3* genes with phylogenetic analysis. (b) Domain architecture of *GhHH3* genes depicting protein motif distribution; Table S1: List of all qPCR primers used in this study. Table S2: Gene ID and proposed names of all observed 19 different plant species including *A. thaliana, B. napus, G. arboreum, G. hirsutum, G. max, G. raimondii, M. truncatula, O. sativa, P. trichocarpa, S. bicolor*, *S. tuberosum, T. cacao*, *V. vinifera*. *Z. mays*, *A. comosus*, *P. taeda*, *C. reinhardtii*, *P. patens*, and *S. moellendorffii;* Table S3: Biophysical properties of *GhHH3* genes including locus ID, start and end point, strand, CDs (coding sequence), protein length, MW (molecular weight), pl (isoelectric point), gravity values, and predicted subcellular localization; Table S4: Genes orthologous/paralogous of in At and Dt sub-genomes of *G. hirsutum*, *G. arboreum* (A genome), and *G. raimondii* (D genome). A total of 81 orthologous/paralogous gene pairs were identified as the result of segmental and whole genome duplication. Further, the Ka/Ks (non-synonymous/synonymous) ratio of all identified orthologous/paralogous gene pairs was calculated; Table S5. Promoter cis-element analysis of 34 *GhHH3* genes. Predicted cis-element in the promoters of *GhHH3* genes were characterized according to their relevance to growth and development, light, and stress responses as well; Table S6. RNA-seq data analysis of 34 *GhHH3* genes in two fuzzless/lintless mutants (*M1l* and *M2l*). Further, genes were categorized on the basis of their up- or downregulated expression in these two mutants.
