# Supplementary figures and images for: Identification of Histone H3 (HH3) Genes in Gossypium hirsutum Revealed Diverse Expression During Ovule Development and Stress Responses
Source: Genes (Basel). 2019 May 9;10(5):355. doi: 10.3390/genes10050355 (PMC6562411; doi:10.3390/genes10050355)

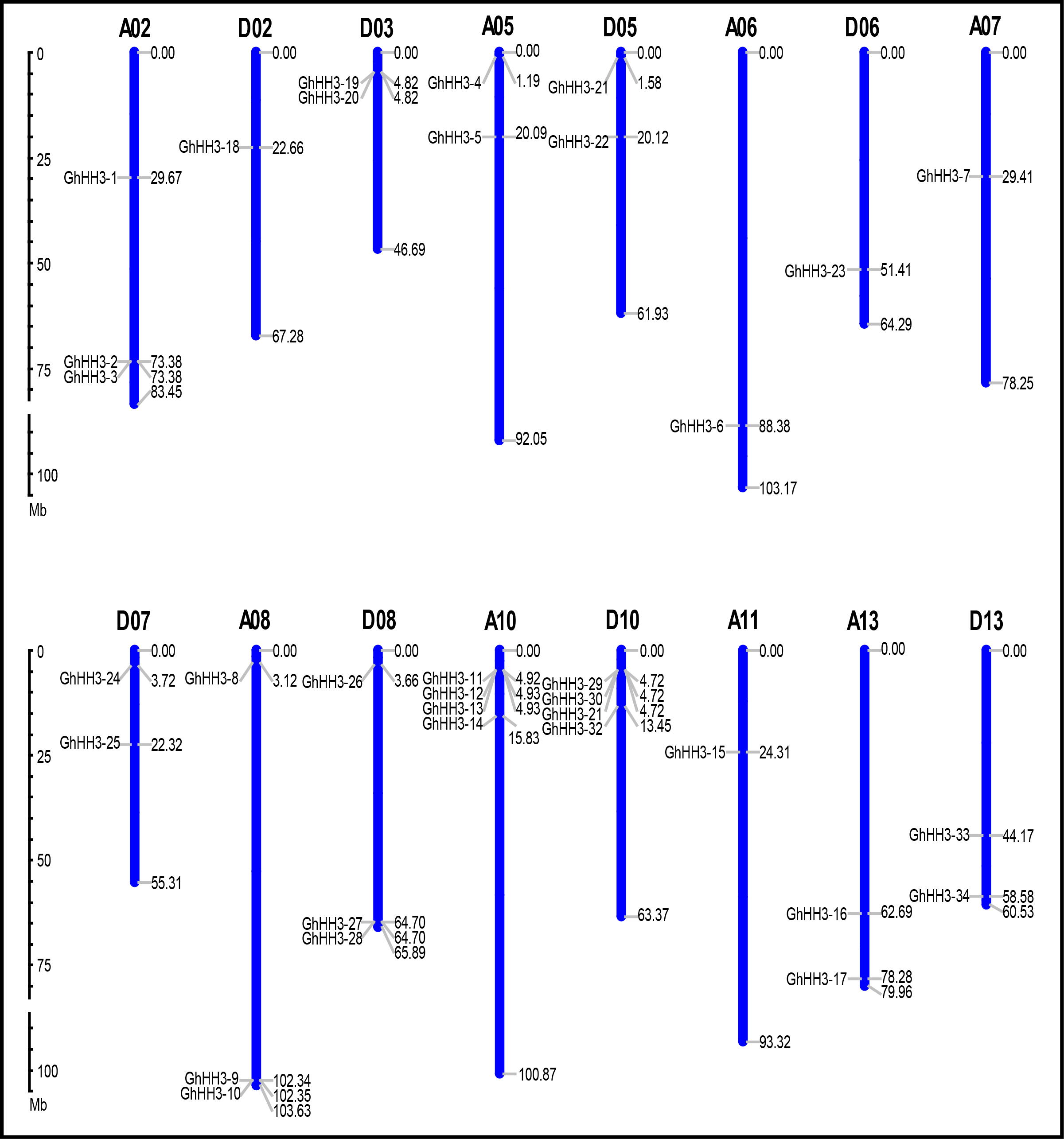

Supplement: Supplementary file 1 [file genes-10-00355-s001.zip › Figure S1.tif]

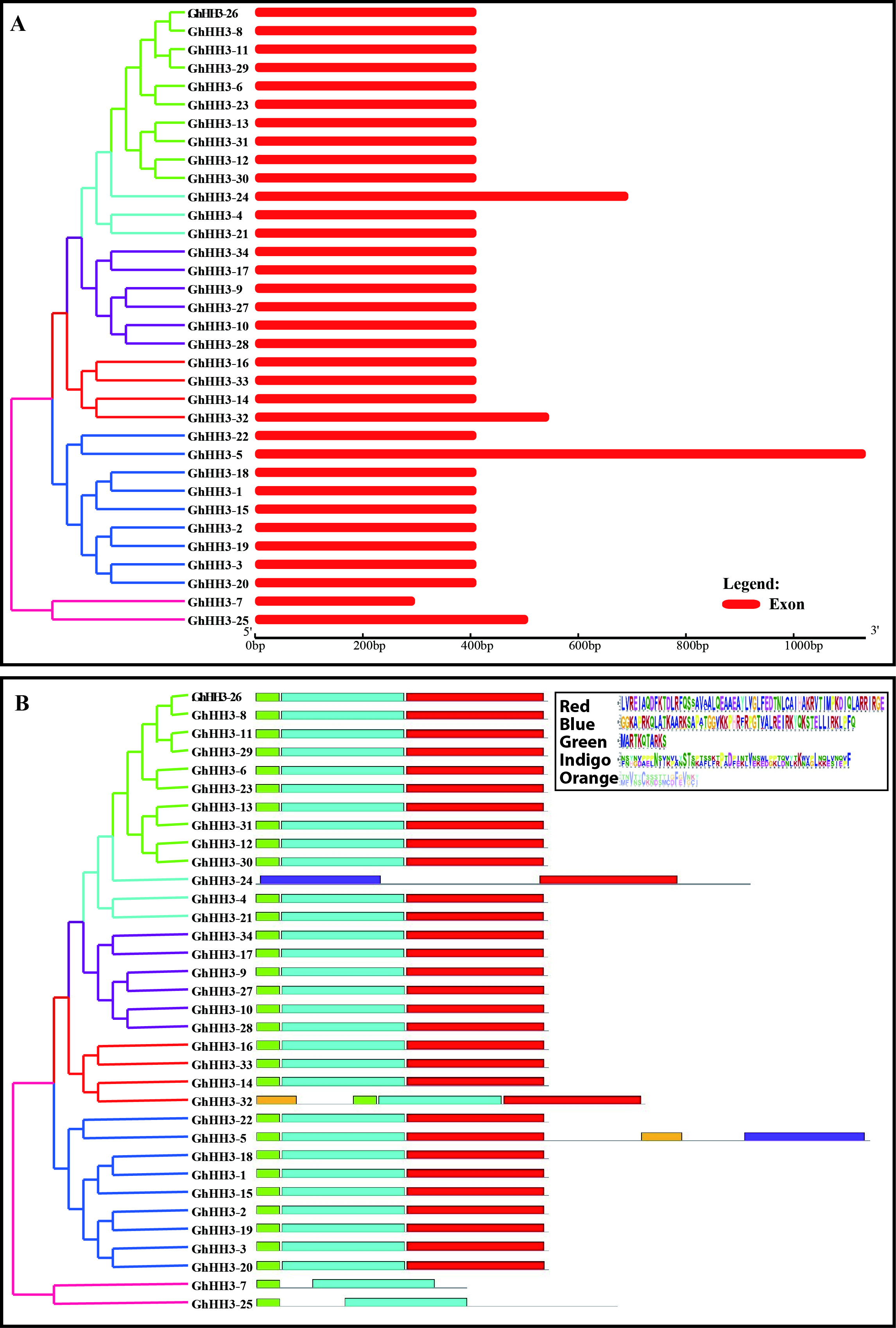

Supplement: Supplementary file 1 [file genes-10-00355-s001.zip › Figure S2.tif]
